# The Circadian Clock Maintains Cardiac Function by Regulating Mitochondrial Metabolism in Mice

**DOI:** 10.1371/journal.pone.0112811

**Published:** 2014-11-12

**Authors:** Akira Kohsaka, Partha Das, Izumi Hashimoto, Tomomi Nakao, Yoko Deguchi, Sabine S. Gouraud, Hidefumi Waki, Yasuteru Muragaki, Masanobu Maeda

**Affiliations:** 1 Department of Physiology, Wakayama Medical University School of Medicine, Wakayama, Japan; 2 First Department of Pathology, Wakayama Medical University School of Medicine, Wakayama, Japan; Karlsruhe Institute of Technology, Germany

## Abstract

Cardiac function is highly dependent on oxidative energy, which is produced by mitochondrial respiration. Defects in mitochondrial function are associated with both structural and functional abnormalities in the heart. Here, we show that heart-specific ablation of the circadian clock gene *Bmal1* results in cardiac mitochondrial defects that include morphological changes and functional abnormalities, such as reduced enzymatic activities within the respiratory complex. Mice without cardiac *Bmal1* function show a significant decrease in the expression of genes associated with the fatty acid oxidative pathway, the tricarboxylic acid cycle, and the mitochondrial respiratory chain in the heart and develop severe progressive heart failure with age. Importantly, similar changes in gene expression related to mitochondrial oxidative metabolism are also observed in C57BL/6J mice subjected to chronic reversal of the light-dark cycle; thus, they show disrupted circadian rhythmicity. These findings indicate that the circadian clock system plays an important role in regulating mitochondrial metabolism and thereby maintains cardiac function.

## Introduction

Heart function relies on oxidative energy that is supplied continuously by mitochondria. Because energy demand in the heart varies across the sleep/wake cycle, timely supply of energy may benefit the heart and optimize its function. This time-dependent energy metabolism is subject to the circadian clock system, in which a set of core clock genes play a major role. Without clock gene function, the daily rhythms in heart energy metabolism are impaired [1], [2]. Interestingly, previous studies have demonstrated that defects in clock gene function not only dampen the metabolic rhythms in the heart but also alter cardiac function [3], indicating a close relationship between clock gene function and heart energy homeostasis with respect to the regulation of cardiac function. However, little is known about how the circadian clock system coordinates the heart energy balance, which directly impacts cardiac function.

As the molecular machinery of the circadian clock was identified, it became clear that the roles of the clock genes were not limited to the regulation of physiological rhythms. Clock genes also participate in fundamental physiological processes, such as the immune response [4], [5], gastrointestinal digestion/absorption [6], [7], and renal function [8], [9]. Perhaps most striking was the observation that even fuel and energy metabolism is under the control of clock genes. Using mouse models of clock gene dysfunction, clock genes were demonstrated to regulate carbohydrate and lipid metabolism, such as hepatic gluconeogenesis [10], [11], pancreatic insulin secretion [12], and fat cell differentiation [13],[14]. In addition to these tissue-specific roles of clock genes in energy metabolism, more ubiquitous metabolic processes, such as the control of cellular redox, are associated with clock-gene function [15], [16], [17]. More importantly, recent studies have revealed that mitochondrial oxidative respiration is also influenced by the function of clock genes in liver and muscle [18], [19]. Because functional clock genes are widespread throughout the body [20], [21], mitochondrial function may be impaired in almost every cell if clock genes do not function appropriately, and this mitochondrial dysfunction may be more apparent in energy-demanding tissues, such as the heart and muscles.

Defects in mitochondrial function have been implicated in the development of heart failure in humans and rodents [22], [23]. Mitochondrial function is highly dependent on the number and/or structure of these organelles and on their capacity for metabolic processes, including fatty acid oxidation (FAO), the tricarboxylic acid (TCA) cycle, and mitochondrial respiration. A substantial portion of the literature has focused on the role of mitochondrial metabolism in the maintenance of cardiac function and has uncovered an integrated regulation of mitochondrial dynamics and bioenergetics by transcription factors and their related molecules [24], [25], [26]. These factors include peroxisome proliferator-activated receptors (PPARs) [27], [28], estrogen-related receptors (ERRs) [29], [30], and PPARγ coactivator-1 (PGC-1) [31], and transcript levels of these genes influence the contractility of cardiac muscle. Importantly, some of these transcription factors interact with protein products of clock genes. Specifically, clock genes and genes encoding PPARα and PGC-1α, key players in mitochondrial dynamics and bioenergetics, interact to regulate their own transcription [32], [33], [34], indicating a close association between the clock and mitochondrial function at the molecular level.

In this study, we hypothesized that cardiac mitochondria may require normal circadian clock function to maintain cardiac function. To demonstrate that dysregulation of the circadian clock leads to cardiac mitochondrial defects, which then leads to reduced cardiac function, we used two mouse models of circadian clock dysregulation, one in which the clock gene *Bmal1* was knocked out in the heart and another that was exposed to continuous disturbance of the external light-dark (LD) cycle, which disrupts behavioral and physiological rhythms.

## Results

### Heart-specific *Bmal1* knockout mice develop congestive heart failure with age

**Figure 1:**
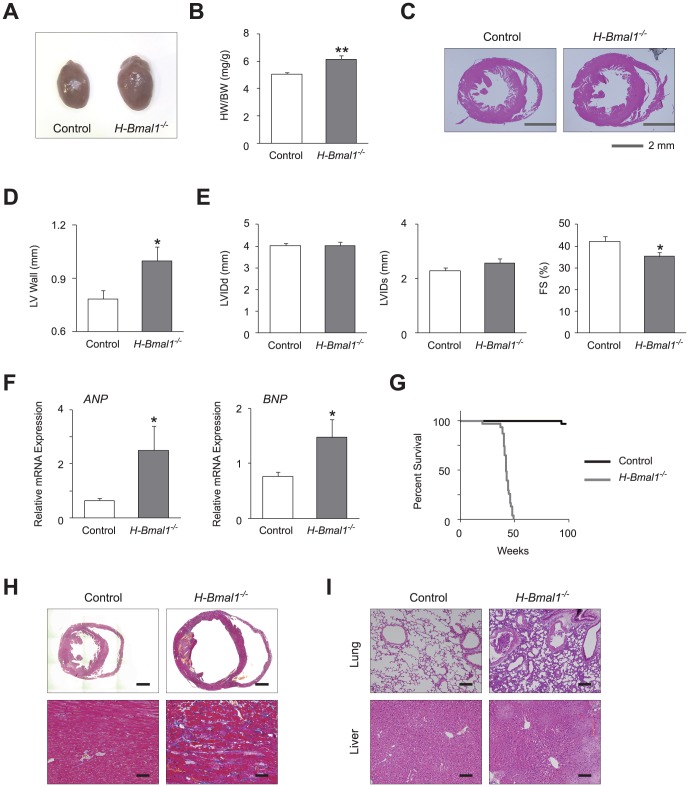
*H-Bmal1* ^−/−^ mice develop progressive congestive heart failure with age. (A) Representative gross morphology of hearts from 12-week-old control and *H-Bmal1* ^−/−^ mice. (B) Ratios of heart weight to body weight (HW/BW) at 12 weeks of age (n = 6 per group). (C) Low-power views after H&E staining of transverse sections from control and *Bmal1* ^−/−^ hearts at 12 weeks of age. (D) LV lateral wall thickness determined in histological images used in (C) (n = 5 per group). (E) Echocardiographic analysis in 12-week-old control and *H-Bmal1* ^−/−^ animals (n = 8–9 per group). LV internal diameter at diastole (LVIDd) and at systole (LVIDs) and fractional shortening (FS) are shown in bar graph format. (F) Transcript expression levels of *ANP* and *BNP* in control and *H-Bmal1* ^−/−^ mice at 12 weeks of age (n = 6 per group). (G) Kaplan-Meier survival curves of control and *H-Bmal1* ^−/−^ animals (n = 31 per group). (H) Low-power views (top panels, scale bar: 1 mm) and high-magnification views (bottom panels, scale bar: 25 µm) of Masson's trichrome staining of heart sections from 33-week-old control and *H-Bmal1* ^−/−^ mice. (I) High-magnification views of H&E staining of lung (top panels) and liver (bottom panels) sections from the same animals used in (H). Scale bar: 50 µm. Data are the mean ± SEM. *P<0.05, **P<0.01, unpaired two-tailed Student's *t*-test.

To test the hypothesis that the circadian clock is involved in the maintenance of cardiac function by regulating mitochondrial metabolism, we first examined the effect of heart-specific disruption of the clock on both cardiac morphology and function. We generated heart-specific *Bmal1* knockout (*H-Bmal1* ^−/−^) mice by crossing mice bearing a floxed *Bmal1* gene with mice expressing the Cre recombinase under the control of the αMHC promoter. We compared the cardiac phenotypes of 12-week-old *H-Bmal1* ^−/−^ mice with those of littermate control animals with a floxed *Bmal1* gene but no *Cre* transgene. As shown in previous reports using mice with heart-specific disruption of clock genes [35], *H-Bmal1* ^−/−^ mice showed an increase in heart size and heart weight (HW)/body weight (BW) ratio compared with control animals (Figure 1, A and B). Histological analysis revealed that the increase in heart size observed in the *H-Bmal1* ^−/−^ mice was due to thickening of the left ventricular (LV) wall (Figure 1, C and D). In addition to these morphological changes, heart function was also altered in *H-Bmal1* ^−/−^ mice. Although neither the LV internal diameter at diastole (LVIDd) nor systole (LVIDs), as assessed by echocardiogram, was significantly different, LVIDs tended to be higher in *H-Bmal1* ^−/−^ mice compared with control animals, which led to a significant decrease in fractional shortening (Figure 1E), indicating that *H-Bmal1* ^−/−^ mice developed heart failure. Consistent with these functional analyses, the expression levels of markers of heart failure (*ANP* and *BNP*) were increased in heart tissue from *H-Bmal1* ^−/−^ mice (Figure 1F). Although cardiac function may have been affected by disrupted physiological rhythmicity, such as behavioral and cardiovascular rhythms, the daily rhythms in locomotor activity, blood pressure, and heart rate were not different between control and *H-Bmal1* ^−/−^ mice (Figure S1, A and B).

We further explored the impact of age on heart failure in *H-Bmal1* ^−/−^ mice by examining 24-week-old or older animals. As was observed in the 12-week-old animals, the HW/BW ratio was increased in 24-week-old *H-Bmal1* ^−/−^ mice, although the degree of the difference between *H-Bmal1* ^−/−^ and control animals was smaller at 24 weeks of age than at 12 weeks of age (Figure 1B and Figure S2A). In contrast, functional analyses showed that *H-Bmal1* ^−/−^ animals at 24 weeks of age exhibited a more profound decrease in heart function than at 12 weeks of age. Although not observed in 12-week-old animals, both LVIDd and LVIDs were significantly increased in 24 week-old *H-Bmal1* ^−/−^ mice compared with control animals (Figure 1E and Figure S2B). Furthermore, systolic contractility (fractional shortening) was more severely decreased in 24-week-old than 12 week-old *H-Bmal1* ^−/−^ mice when compared with age-matched control animals (16% vs. 20% decrease at 12 and 24 weeks of age, respectively; Figure 1E and Figure S2B).

The progressive reduction in heart function with age resulted in early mortality in *H-Bmal1* ^−/−^ mice. Although almost all control animals survived beyond the study period (96 weeks), the majority of *H-Bmal1* ^−/−^ mice died at approximately 40 weeks of age (average life span, control 95.8 weeks vs. *H-Bmal1* ^−/−^ 41.3 weeks; log-rank test, χ^2^ = 62.0, df = 1, p<0.001; Figure 1G). The existence of quite severe heart failure was indicated in histological analyses from 33-week-old *H-Bmal1* ^−/−^ mice, which showed ventricular dilation, thinned myocardial walls, thrombosis in the ventricle, and myocardial fibrosis (Figure 1H). In addition to the heart tissue, histological changes that indicated severely decreased cardiac function were observed in other organs of 33-week-old *H-Bmal1* ^−/−^ mice. The lung tissue of these animals showed vascular wall thickness, congestion, and infiltration of inflammatory cells, which are all often observed with severe congestive heart failure (Figure 1I). Signs of congestion in *H-Bmal1* ^−/−^ animals were also observed in the liver, including dilatation of hepatic sinusoids and central veins (Figure 1I). Collectively, our data show that defects in the function of the *Bmal1* gene in heart lead to progressive heart failure, which results in congestion of multiple organs and a short life span.

### *Bmal1* deficiency alters cardiac energy metabolism

**Figure 2:**
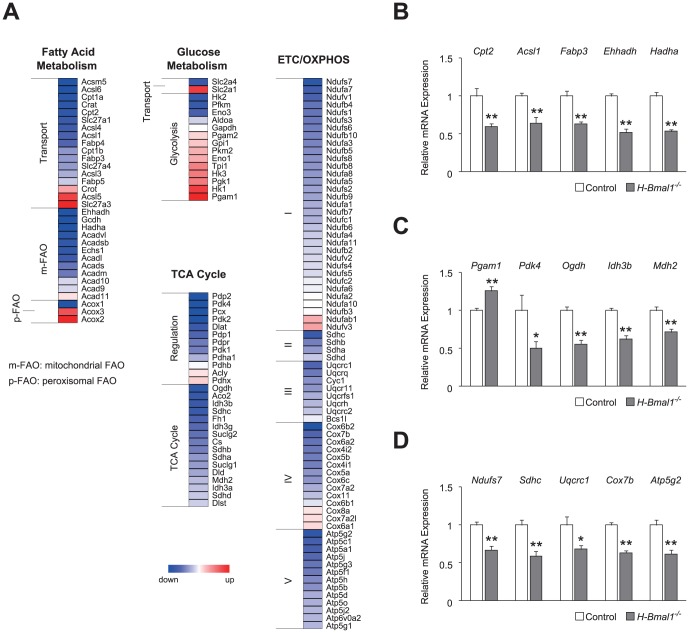
Gene expression profiles of cardiac energy metabolism in *H-Bmal1* ^−/−^ mice. (A) Heat-map representations of several classes of metabolic genes detected in the expression analysis of *Bmal1* ^−/−^ hearts from 12-week-old animals. (B-D) Relative expression levels of representative genes regulating (B) fatty acid metabolism, (C) glycolysis/TCA cycle, and (D) ETC/OXPHOS are shown (n = 6 per group). Data are the mean ± SEM. *P<0.05, **P<0.01, unpaired two-tailed Student's *t*-test (B-D).

To examine whether the heart failure observed in *H-Bmal1* ^−/−^ mice was due to alterations in cardiac energy metabolism, we performed a DNA microarray analysis to detect changes in expression of the genes responsible for this failure. As approximately 8% of the genes expressed in the heart show circadian rhythmicity [36], and because we only used samples dissected at ZT 2 (ZT 0 defined as lights on), the limitations of our microarray analysis are that the detected changes might simply be due to shifted peaks or troughs of gene expression and that the analysis might not detect all gene expression changes. Nevertheless, we found that broad classes of genes regulating cellular energy metabolism were upregulated or downregulated in the heart tissues of 12-week-old *H-Bmal1* ^−/−^ mice compared with those of control animals (Figure 2A). Because fatty acids are the preferred metabolic substrate for adult cardiomyocytes, we first investigated the expression of genes regulating fatty acid metabolism. Although a few genes were upregulated, the majority of genes associated with fatty acid transport were downregulated in *Bmal1* ^−/−^ hearts (Figure 2A). In addition to fatty acid transporters, the mRNA expression levels of a variety of enzymatic components that regulate mitochondrial FAO (e.g., *Ehhadh* and *Hadha*) were also downregulated in *Bmal1* ^−/−^ hearts (Figure 2A). Importantly, despite the decrease in mitochondrial FAO genes, genes for peroxisomal FAO (*Acox 2* and *Acox3*) were upregulated in *Bmal1* ^−/−^ hearts (Figure 2A), indicating some compensation from mitochondrial to peroxisomal FAO. Validation analyses using quantitative PCR also revealed a significant decrease in the expression levels of *Cpt2*, *Acsl1* (the rate-limiting enzyme for the activation of fatty acids), *Fabp3*, *Ehhadh*, and *Hadha* (the rate-limiting enzyme for mitochondrial FAO) in *Bmal1* ^−/−^ hearts (Figure 2B).

Because glucose metabolism in cardiomyocytes takes on greater importance under pathologic conditions including myocardial ischemia and hypertrophy, we next investigated the possibility of compensatory induction of the glycolytic pathway in *Bmal1* ^−/−^ hearts. Indeed, in *H-Bmal1* ^−/−^ mice, the expression of the glucose transporter, which mediates basal glucose uptake into cardiomyocytes (*Slc2a1*, also known as *Glut1*), was upregulated, although the insulin-dependent glucose transporter (*Slc2a4*, also known as *Glut4*) was downregulated (Figure 2A). In addition, 9 of 14 glycolytic genes examined were upregulated in *Bmal1* ^−/−^ hearts (Figure 2A). Quantitative PCR validation analyses revealed that *Pgam1* (a glycolytic enzyme) was significantly increased and that *Pdk4* (a negative regulator of glucose oxidation) was decreased by approximately 50% in *Bmal1* ^−/−^ hearts (Figure 2C). Collectively, these data indicate that the energy substrate preference is altered in *Bmal1* ^−/−^ hearts.

We next examined the expression of genes controlling TCA cycle and the electron transport chain (ETC)/oxidative phosphorylation (OXPHOS), common downstream pathways for both FAO and glycolysis. In contrast to the expression of FAO and glycolytic genes, which showed both upregulation and downregulation, the expression levels of the enzymatic components of TCA cycle and ETC/OXPHOS pathway were generally downregulated in *Bmal1* ^−/−^ hearts (Figure 2A). In particular, within TCA cycle, the genes encoding subunits of the enzymes that reduce NAD^+^ to NADH (*Ogdh*, *Idh3b*, and *Mdh2*) were significantly decreased in *Bmal1* ^−/−^ hearts (Figure 2, A and C). Similar to TCA cycle, the majority of genes in the ETC/OXPHOS pathway were downregulated in *Bmal1* ^−/−^ hearts (Figure 2A). The significant decreases in representative genes (*Ndufs7*, *Sdhc*, *Uqcrc1*, *Cox7b*, and *Atp5g2*) associated with the ETC/OXPHOS pathway were also validated by quantitative PCR (Figure 2D).

### Defects in *Bmal1* function alter mitochondrial dynamics in heart

**Figure 3:**
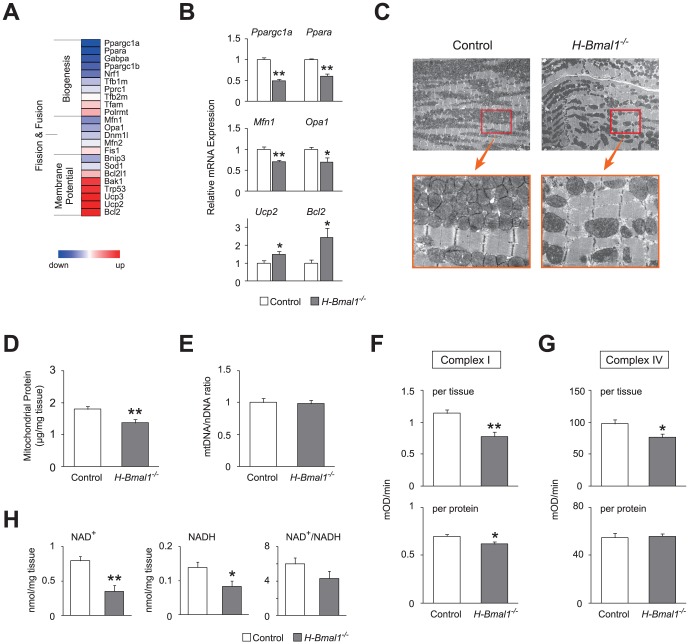
Mitochondrial abnormalities in the hearts of 12-week-old *H-Bmal1* ^−/−^ mice. (A) A heat-map representing the expression profiles of genes regulating mitochondrial structure and function in *Bmal1* ^−/−^ hearts. (B) Relative expression levels of genes associated with mitochondrial biogenesis, dynamics, and membrane potential in the heart tissue of control and *H-Bmal1* ^−/−^ animals (n = 6 per group). (C) Representative electron micrographs of sections taken from the left ventricular muscle from control and *H-Bmal1* ^−/−^ mice at two different magnifications. (D) Mitochondrial protein concentration in control and *Bmal1* ^−/−^ hearts (n = 8 per group). (E) Mitochondrial DNA to nuclear DNA ratio in control and *Bmal1* ^−/−^ hearts (n = 6 per group). (F-G) Enzymatic activities of (F) complex I and (G) complex IV in control and *Bmal1* ^−/−^ hearts (n = 8 per group). The activities of the mitochondrial respiratory enzymes are expressed either per milligram of tissue used for mitochondrial isolation (top panels) or per microgram of mitochondrial protein (bottom panels). (H) NAD^+^ and NADH concentrations in control and *Bmal1* ^−/−^ hearts (n = 6 per group). Data are the mean ± SEM. *P<0.05, **P<0.01, unpaired two-tailed Student's *t*-test.

Because loss of *Bmal1* function induced a decrease in the expression of a broad range of genes controlling mitochondrial energy metabolism, we next examined the expression of transcripts that are associated with mitochondrial dynamics in *Bmal1* ^−/−^ hearts. Microarray analyses revealed that *Bmal1* deficiency downregulated a variety of genes controlling mitochondrial biogenesis as well as fission and fusion in the heart (Figure 3A). Validation analyses revealed that *Ppargc1a* (also known as PGC-1α) and *Ppara* (also known as PPARα), which are the genes responsible for mitochondrial biogenesis, were decreased significantly by 50% and by 40%, respectively (Figure B). In addition, a significant reduction was also found in genes associated with mitochondrial quality control (*Mfn1 and Opa1;* Figure 3B). Although these mitochondrial structure-related genes were downregulated, genes associated with the loss of mitochondrial membrane potential were upregulated in *Bmal1* ^−/−^ hearts (Figure 3A). Specifically, genes controlling the permeability of the mitochondrial membrane (*Ucp2* and *Bcl2*) were significantly increased in the heart tissue of *H-Bmal1* ^−/−^ mice (Figure 3B). These results suggest that *Bmal1* ^−/−^ hearts were defective in both the regulation of mitochondrial structure and membrane potential at the gene expression level.

Consistent with the downregulation of genes controlling mitochondrial dynamics, electron microscopic analysis showed morphological changes in the mitochondria of *Bmal1* ^−/−^ hearts. In heart tissue from 12-week-old *H-Bmal1* ^−/−^ mice, small- and large-sized mitochondria coexisted, whereas control hearts showed almost identical sizes (Figure 3C). In addition, the relative number of mitochondria was also significantly decreased in *Bmal1* ^−/−^ hearts (control 1.00±0.03 vs. *Bmal1* ^−/−^ 0.70±0.05; p<0.01). Ultrastructural examinations showed that mitochondria were sparse in *Bmal1* ^−/−^ hearts, whereas control hearts showed a dense mitochondrial population (Figure 3C). This observation was consistent with our finding that mitochondrial protein levels extracted from *Bmal1* ^−/−^ hearts were significantly reduced compared with those from control hearts (Figure 3D). Similar changes in both the size and the number of mitochondria were also observed in heart tissue from 24-week-old *H-Bmal1* ^−/−^ mice (Figure S3, A and B).

### Hearts from *H-Bmal1^−/−^* mice showed altered mitochondrial function at the genomic and biochemical levels

In addition to the morphological examination, we also conducted functional analyses of cardiac mitochondria in *H-Bmal1* ^−/−^ mice. We first examined the ratio of mitochondrial DNA (mtDNA) to nuclear DNA (nDNA) copy number because the mtDNA copy number can vary independently of nDNA or mitochondrial number, and this ratio serves as a marker of individual mitochondrial function at the organelle level. Despite the decrease in the number of mitochondria in *Bmal1* ^−/−^ hearts (Figure 3, C and D), the ratio of mtDNA to nDNA was not different between 12-week-old control and *H-Bmal1* ^−/−^ animals (Figure 3E), which likely indicates compensation by increasing the copy number of mtDNA per mitochondrion in *Bmal1* ^−/−^ hearts. In contrast to younger animals, *Bmal1* ^−/−^ hearts from 24-week-old animals showed an approximate 30% decrease in the mtDNA/nDNA ratio compared with control hearts (Figure S3C).

We next examined biochemical activities of the ETC complexes in *Bmal1* ^−/−^ hearts. We analyzed complex I activity because the circadian clock links to redox metabolism [15], [16], [17], in which complex I accounts for almost all oxidative conversion from NADH to NAD^+^ in the cell. Consistent with our microarray analyses, which showed an overall decrease in the expression of complex I genes (Figure 2A), complex I activity was significantly decreased in *Bmal1* ^−/−^ hearts (Figure 3F). Importantly, this result was not only observed when analyzed using the same weight of heart tissues (Figure 3F, top) but was also detected when normalized to mitochondrial protein concentration (Figure 3F, bottom), indicating that complex I activity in individual mitochondria from *Bmal1* ^−/−^ hearts was indeed attenuated. Complex IV activity was also significantly decreased in heart tissue from 12-week-old *H-Bmal1* ^−/−^ mice when normalized to tissue weight (Figure 3G, top). However, when normalized to mitochondrial protein concentration, complex IV activity was almost identical between control and knockout animals (Figure 3G, bottom), which indicates that the decrease in complex IV activity was not caused by reduced enzymatic activity in each mitochondrion but due to the decreased number of mitochondria in *Bmal1* ^−/−^ hearts. A similar but more profound reduction in complex I and IV activities was also observed in heart tissue from 24-week-old *H-Bmal1* ^−/−^ animals (Figure S3, D and E).

We also examined the levels of NAD^+^ and NADH in *Bmal1* ^−/−^ hearts because the intracellular redox state is tightly coupled to enzymatic activities of ETC complexes. In 12-week-old animals, both NAD^+^ and NADH levels were dramatically decreased by approximately 56% and 40%, respectively, in *Bmal1* ^−/−^ hearts relative to control hearts (Figure 3H). The NAD^+^/NADH ratio in *Bmal1* ^−/−^ hearts was also decreased, although this difference did not reach statistical significance (Figure 3H). In 24-week-old *H-Bmal1* ^−/−^ animals, we also observed similar but more severe changes in NAD^+^ and NADH levels (approximately 78% and 83% reductions, respectively, Figure S3F). Collectively, these findings suggest that the *Bmal1* gene is essential for mitochondrial metabolism in the heart and that loss of *Bmal1* function causes progressive defects in cardiac mitochondrial structure and function with age.

### Loss of *Bmal1* function induces the expression of genes related to cardiac remodeling

**Figure 4:**
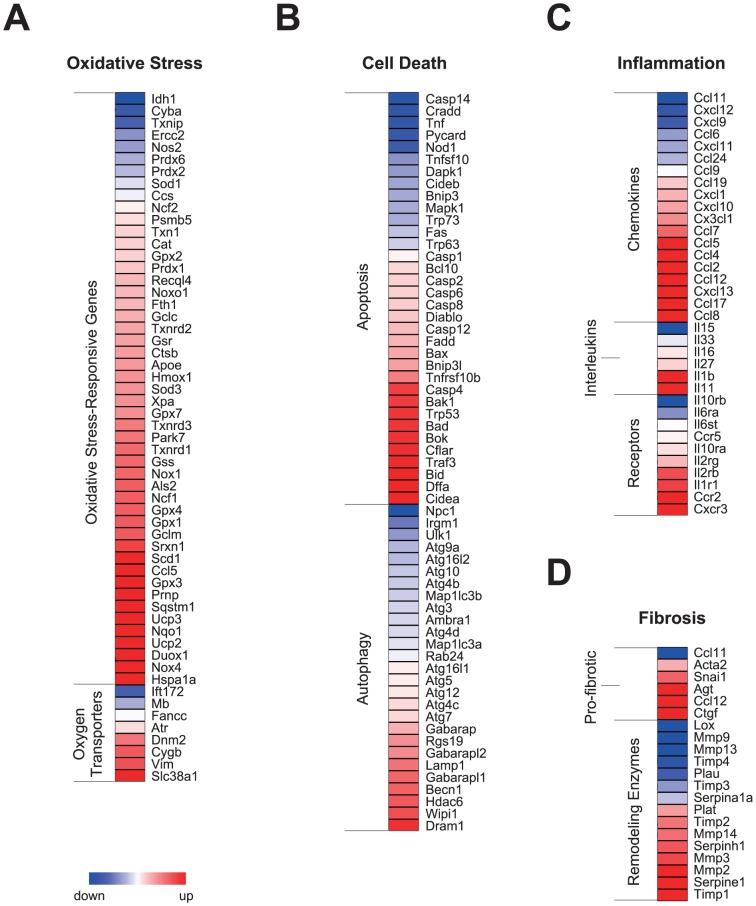
Expression profiles of genes associated with pathologic ventricular remodeling in *Bmal1* ^−/−^ hearts. (A-D) Heat-map representations of genes involved in (A) the response to oxidative stress, (B) programmed cell death, (C) inflammation, and (D) fibrosis in hearts from 12-week-old *Bmal1* ^−/−^ mice.

Because cardiac remodeling processes are often observed in failing hearts, we next explored whether genes associated with cardiac remodeling were increased in *Bmal1* ^−/−^ hearts. We examined the expression levels of genes related to oxidative stress, programmed cell death, inflammation, and fibrosis, which are common cellular changes observed in remodeling hearts. Our microarray analyses revealed that the majority of oxidative stress-responsive genes (e.g., *Gpx* and *Sod*) and genes responsible for oxygen transport (e.g., *Slc38a1* and *Cygb*) were upregulated in *Bmal1* ^−/−^ hearts (Figure 4A), indicating the existence of oxidative stress in heart tissue from *H-Bmal1* ^−/−^ animals. Increased oxidative stress can lead to programmed cell death. We observed an upregulation of genes important for the induction of apoptosis and autophagy (Figure 4B). These genes included those encoding caspases (*Casp*), proapoptotic homologs of members of the Bcl-2 family (*Bax*, *Bak*, *Bad* and *Bid*), and autophagy-associated genes (e.g., *Dram1* and *Becn1*).

In addition, the mRNA expression levels were upregulated for a variety of inflammatory genes, such as genes encoding chemokines and their receptors (e.g., *Ccl2*, also known as *MCP-1*, and its receptor *Ccr2*) as well as interleukin-1 beta (*Il1b*), in *Bmal1* ^−/−^ hearts (Figure 4C). Furthermore, consistent with the histological analysis, which showed fibrotic changes (Figure 1H), genes responsible for pro-fibrotic changes (e.g., *Ctgf* and *Snai1*) and remodeling enzyme genes (e.g., *Timp* and *Serpine1*) were also increased in heart tissue from *H-Bmal1* ^−/−^ mice (Figure 4D). These results indicate that *Bmal1* ^−/−^ hearts undergo pathologic ventricular remodeling at the gene expression level.

### Disruption of circadian behaviors reduces cardiac function in C57BL/6J mice with drug-induced cardiomyopathy

**Figure 5:**
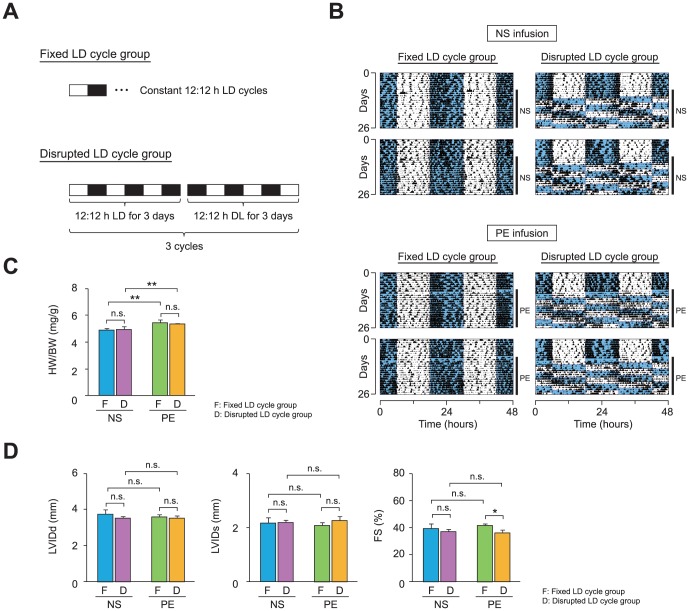
Circadian desynchronization reduces cardiac function in C57BL/6J mice with drug-induced cardiomyopathy. (A) A light-dark (LD) cycle regimen used to examine the effects of a variable LD schedule on cardiac function. C57BL/6J mice were either maintained on a constant LD schedule (fixed LD cycle group) or were subjected to a 12-h phase shift in LD cycle every 3 days (disrupted LD cycle group). To compare the effects of the LD schedule between animals with healthy hearts and those with cardiomyopathy, either normal saline (NS) or phenylephrine (PE) was continuously infused via an osmotic pump in each LD cycle group. (B) Locomotor activity records of NS-infused (top panel) and PE-infused (bottom panel) mice. Two representative records from animals subjected to the fixed (left column) and disrupted (right column) LD cycle are shown. Activity counts are indicated by the vertical black marks. The records are double-plotted such that 48 h are shown for each horizontal trace. The blue shaded and unshaded areas indicate the dark and light period, respectively. The duration of NS or PE infusion is indicated by the vertical line at the right margin. (C) The ratios of heart weight to body weight (HW/BW) were increased by the PE infusion but were not influenced by the disruption in LD cycle (n = 5–8 per group). (D) The effects of PE, disrupted LD cycle, or both on ventricular function were evaluated using echocardiographic measurements (n = 5–8 per group). LVIDd, LVIDs, and FS are shown in bar graph format. Data are the mean ± SEM. *P<0.05, **P<0.01, two-way ANOVA.

Having demonstrated that mitochondrial metabolism is disrupted in *Bmal1*-null hearts, which is accompanied by pathologic cardiac remodeling, we sought to examine whether cardiac mitochondrial metabolism is also impaired by the circadian disorder induced by disturbing the external LD cycle. Previous studies have shown that continuously shifting the LD and/or sleep/wake cycle is associated with increased cardiovascular morbidity or mortality in both humans and animals [37], [38]. These observations, together with our findings in *H-Bmal1* ^−/−^ animals, raise the possibility that chronic desynchronization of the internal circadian clock with the external LD cycle, a condition often observed in shift work, may reduce cardiac function by altering mitochondrial metabolism in the heart. To test this hypothesis, we first examined the cardiac phenotypes of C57BL/6J mice either maintained on a fixed 12∶12 h LD cycle (fixed LD cycle group) or exposed to a 12-h phase shift in the LD cycle every 3 days (disrupted LD cycle group) for 18 days (Figure 5A). To compare the effects of our disrupted LD cycle regimen between animals with healthy hearts and those with pathologic hearts, C57BL/6J mice were infused either with normal saline (NS) as a vehicle control or with phenylephrine (PE), a hypertrophic stimulus, via an osmotic pump. Infrared-based detection of locomotor activity revealed that the temporal pattern of locomotor activity was sensitive to and followed the chronic shifting of the LD cycle in both NS-infused and PE-infused animals (Figure 5B), indicating that our LD reversal protocol was effective in disrupting circadian behaviors.

To examine the effects of the disrupted LD cycle on cardiac phenotypes, we first compared changes in heart morphology between the control and experimental groups. As expected, the PE infusion increased the HW/BW ratio significantly when compared with vehicle infusion in both fixed and disrupted LD cycle groups (Figure 5C). In addition, although not statistically significant, the LV posterior diameter in diastole, as assessed by echocardiogram, was also increased in PE-infused animals (NS-infused 0.92±0.11 mm vs. PE-infused 1.12±0.10 mm). Although these results indicate that PE infusion induced cardiac hypertrophy, the disruption in LD cycle did not further affect heart weight in PE-infused animals (Figure 5C).

We next investigated whether the disrupted LD cycle altered cardiac function. Interestingly, although the disrupted LD cycle did not alter the weight of hearts (Figure 5C), heart function was significantly reduced in PE-infused but not NS-infused animals when subjected to the disrupted LD cycle (Figure 5D). Specifically, in PE-infused mice, the disrupted LD cycle significantly decreased fractional shortening, as assessed by echocardiogram, although we found no difference in LVIDd and a small, non-significant increase in LVIDs (Figure 5D). In NS-infused animals, although not statistically significant, systolic contractility (fractional shortening) tended to be lower when mice were exposed to the disrupted LD cycle (Figure 5D). These results suggest that exposure to disrupted LD cycles adversely affects cardiac function in mice.

### Disruption of circadian behaviors not only alters rhythms but also reduces levels of transcripts associated with energy metabolism in the heart

**Figure 6:**
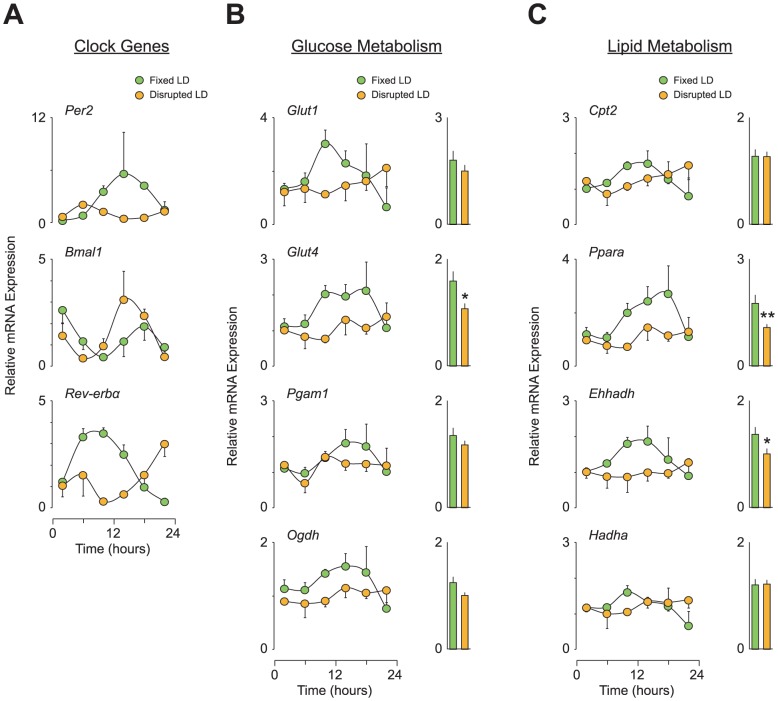
Circadian desynchronization not only disrupts rhythms but also reduces the expression levels of clock and metabolic genes in the heart of C57BL/6J mice with PE-induced cardiomyopathy. (A-C) Relative expression levels of genes regulating (A) clock machinery as well as (B) glucose and (C) lipid metabolism in heart. All heart tissues used were from PE-infused animals subjected to either a fixed or a disrupted LD cycle as described in Figure 5A (n = 4 per group per time point). To provide a 24-h overall mean expression level, the data over a 24-h time period in each group were also averaged and are expressed using a bar graph format. Data are the mean ± SEM. *P<0.05, **P<0.01, unpaired two-tailed Student's *t*-test.

To determine the genetic basis for the reduced cardiac function caused by the disrupted LD cycle, we examined both the rhythms and expression levels of circadian and metabolic genes in heart tissue from NS-infused and PE-infused C57BL/6J mice. Under the fixed LD cycle condition, all three clock genes examined (*Per2*, *Bmal1*, and *Rev-erbα*) displayed clear diurnal rhythms in expression levels in both NS-infused and PE-infused animals (Figure S4A and Figure 6A, respectively). However, in both NS-infused and PE-infused animals, the time of peak expression of these clock genes was shifted when mice were exposed to a disrupted LD cycle (Figure 6A and Figure S4A). Furthermore, interestingly, the *Per2* gene displayed a decrease in expression levels throughout the day when under the disrupted LD cycle condition.

We also found that the disrupted LD cycle altered the expression of metabolic genes. Under the fixed LD cycle regimen, the glucose transporter genes (*Glut1* and *Glut4*), a glycolytic gene (*Pgam1*), and a gene within TCA cycle (*Ogdh*) all exhibited diurnal variations in their expression in heart tissue from both NS-infused and PE-infused mice (Figure S4B and Figure 6B, respectively). Similarly, expression levels of genes associated with fatty acid transport (*Cpt2*) and β oxidation (*Ppara*, *Ehhadh*, and *Hadha*) also displayed diurnal patterns when exposed to the fixed LD cycle condition (Figure 6C and Figure S4C). Under the disrupted LD cycle, we expected phase-shifted expression patterns of these metabolic genes, as observed with clock gene expression; however, interestingly, these metabolic genes showed changes in their expression levels (i.e., downregulation) rather than altered expression rhythms when the animals were subjected to the disrupted LD cycle (Figure 6, B and C, Figure S4, B and C). In particular, when overall 24-h expression levels were averaged, a significant decrease was observed in *Glut4*, *Ppara*, and *Ehhadh* in PE-infused animals (Figure 6, B and C) and in *Glut1* and *Ppara* in NS-infused mice (Figure S4, B and C).

### Chronic circadian desynchronization disrupts mitochondrial metabolism in the heart

**Figure 7:**
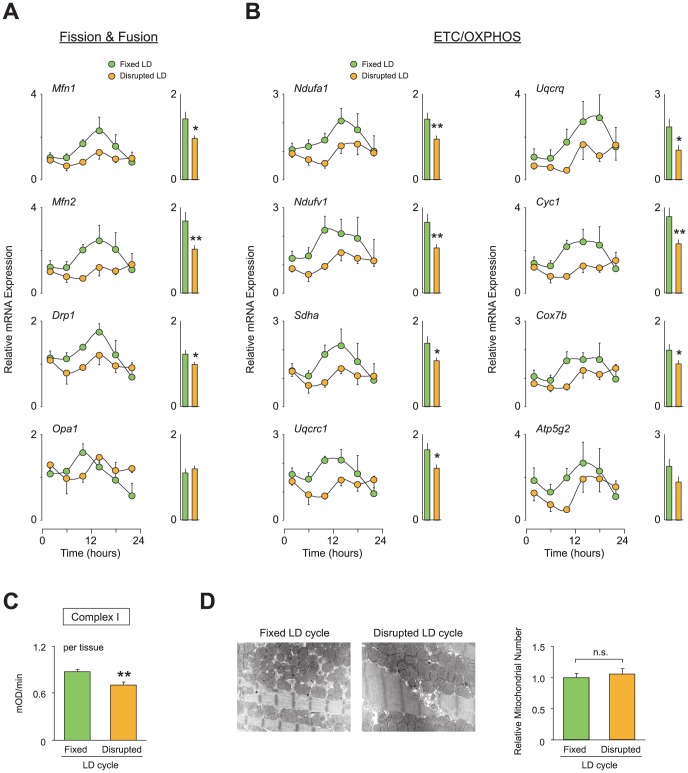
Circadian desynchronization impairs mitochondrial function in the hearts of C57BL/6J mice with PE-induced cardiomyopathy. (A-B) Relative expression levels of genes regulating (A) mitochondrial structure or (B) mitochondrial oxidative metabolism in heart. All heart tissues used are from PE-infused animals subjected to either a fixed or a disrupted LD cycle as described in Figure 5A (n = 4 per group per time point). To provide a 24-h overall mean expression level, the data over a 24-h time period in each group were also averaged and are expressed in bar graph format. (C) Enzymatic activity of complex I in PE-infused animals exposed to a fixed or disrupted LD cycle (n = 4–5 per group). The complex I activity is expressed per milligram of tissue used for mitochondrial isolation. (D) The relative number of mitochondria in the left ventricular muscle was counted using electron microscope images. Representative images are shown. Data are the mean ± SEM. *P<0.05, **P<0.01, unpaired two-tailed Student's *t*-test.

We next examined whether the disrupted LD cycle also altered cardiac mitochondrial metabolism. We analyzed the temporal expression levels of genes involved in mitochondrial structure (fission and fusion) and function (ETC/OXPHOS pathway). Under the fixed LD cycle, fission and fusion genes (*Mnf1*, *Mfn2*, *Drp1*, and *Opa1*) displayed diurnal variations in expression levels in heart tissues from both NS-infused and PE-infused animals (Figure S5A and Figure 7A, respectively). These time-dependent expression patterns were also observed in genes encoding components of the ETC/OXPHOS in tissue from mice exposed to the fixed LD cycle (Figure 7B and Figure S5B). However, when animals were exposed to the disrupted LD cycle, diurnal variations in the expression of genes regulating mitochondrial structure and function were dampened in both NS-infused and PE-infused animals (Figure 7, A and B, Figure S5, A and B). Importantly, the dampened expression rhythms in these mitochondrial-related genes were more profound in PE-infused animals than in NS-infused mice. Specifically, the overall 24-h expression levels of 10 of 12 examined genes were significantly decreased in PE-infused mice (Figure 7, A and B), whereas significant decreases were detected only in 7 of 12 genes in NS-infused animals (Figure S5, A and B), indicating that the regulation of gene expression associated with mitochondrial metabolism is more susceptible to disrupted LD cycles in pathologic hearts compared with healthy hearts. This idea is further supported by our observation that the disrupted LD cycle significantly reduced the enzyme activity of complex I only in PE-infused animals (Figure 7C) but not in NS-infused animals (Figure S5C). It should be noted that, although we observed dampened expression rhythms of genes regulating mitochondrial dynamics in heart tissue from animals exposed to the disrupted LD cycle (Figure 7A and Figure S5A), ultrastructural examinations did not show a difference in mitochondrial morphology between animals subjected to the fixed and disrupted LD cycles (Figure 7D and Figure S5D), indicating that the altered mitochondrial metabolism induced by disrupted LD cycles was not due to an alteration in the number of mitochondria.

## Discussion

### Heart *Bmal1* is an important component for the maintenance of cardiac function

Our findings that *H-Bmal1* ^−/−^ mice develop severe, progressive heart failure with age and display a markedly shorter life span, likely due to cardiac decompensation, indicate that *Bmal1* gene expression in the heart is an important component that maintains normal cardiac function throughout life. Of note, we did not find changes in rhythms in locomotor activity and blood pressure in *H-Bmal1* ^−/−^ animals, suggesting that reduced function of *Bmal1* ^−/−^ in the heart is not secondary to alterations in behavioral and/or cardiovascular rhythms but instead is primary to cardiac *Bmal1* dysfunction. Although similar cardiac phenotypes have been shown in previous reports using whole-body *Bmal1* knockout mice [3], our findings strengthen the molecular evidence that *Bmal1* in the heart is indeed required to regulate cardiac function because whole-body *Bmal1* knockout animals exhibited various phenotypic disorders that may affect cardiac function, such as dampened diurnal rhythm in blood pressure and metabolic disorders [11], [12], [39].

### Defects in the circadian clock system impair cellular energy metabolism in the heart

Although defects in the function of clock genes other than the *Bmal1* gene also affect the structure and/or the function of the heart [1], [35], the precise molecular mechanisms that connect the circadian clock system to cardiac function have not yet been fully delineated. Recently, the regulation of cardiac electrophysiology has been reported to be controlled by clock genes [40], indicating that the primary function of cardiomyocytes is linked to the circadian clock system. Herein, we demonstrate that the expression levels of broad classes of genes that regulate fundamental cellular metabolism, such as fatty acid and glucose oxidation, are altered in *Bmal1* ^−/−^ hearts. In addition, we observed that heart tissue from C57BL/6J mice exposed to a disrupted LD cycle showed a significant decrease in the 24-h overall expression levels of genes that are essential for glucose and lipid metabolism, suggesting that the circadian clock system (i.e., the internal clock and the external LD cycle, which together coordinate physiological processes) is required to regulate cellular energy metabolism in the heart. These findings are consistent with previous studies demonstrating that fat metabolism in the heart is impaired in *Clock* mutant animals [2], [41].

Using mouse genetic models, a considerable link between the molecular clock machinery and energy metabolism has been shown, particularly in metabolic organs including fat and liver [42], [43], [44], [45]. For example, fat deposition is increased in the adipose tissue of mice carrying a mutant *Clock* gene [46]. Dysregulation of hepatic glucose metabolism has been demonstrated in mice without *Bmal1* function [10], [11]. Although it is not yet clear how the molecular clock participates in the regulation of glucose and lipid metabolism, transcription factors and their related molecules have been proposed as key components that link the circadian and metabolic systems [42]. PPARα, a key transcriptional regulator of FAO binds to the *Bmal1* promoter [33]. The transcription of the gene encoding PPARα is in turn activated by the BMAL1 protein together with its heterodimerized partner CLOCK [34], suggesting a reciprocal regulation of gene expression between circadian and metabolic transcription factors. PGC-1α, a major regulator of mitochondrial biogenesis and respiration, also interacts with a gene regulatory network of the circadian clock [32]. We found that expression levels of both the *Ppara* and *Ppargc1a* genes, which encode PPARα and PGC-1α, were significantly downregulated in *Bmal1* ^−/−^ hearts. Furthermore, a significant decrease of *Ppara* expression was also observed in heart tissue from C57BL/6J animals subjected to chronic reversal of the LD cycle. These results indicate that circadian disorder imposed either by the deletion of the core clock gene or by a disruption of the LD cycle may impair cellular energy metabolism in the heart due to alterations in the regulation of these metabolic transcription factors.

Because the dysregulation of transcription factors has been implicated in heart failure pathogenesis, our observation that the expression levels of metabolic transcription factors are decreased in *Bmal1* ^−/−^ hearts provides insight into the basis for reduced cardiac function in *H-Bmal1* ^−/−^ animals. Among a series of transcription factors, the dysregulation of PPARs, ERRs, and their transcriptional coactivators is involved in the progression of heart failure. Altered function of PPARα induces cardiac hypertrophy in mice [27], [28]. ERRs participate in the regulation of genes associated with cardiac function not only in adult hearts but also in postnatal hearts [29], [30]. In addition, deletion of the gene encoding PGC-1α alters contractile function of cardiac muscle [31]. Our findings that *Ppara* and *Ppargc1a* were downregulated in *Bmal1* ^−/−^ hearts provide additional evidence that circadian and metabolic transcription factors coordinate a gene regulatory network that affects cardiac performance.

### The circadian clock is required for mitochondrial dynamics and bioenergetics in the heart

Increasing evidence suggests that dysregulation of metabolic transcription factors, such as PPARα and PGC-1α, underlie the defects in mitochondrial structure and function in the failing heart. An alteration in transcriptional control by PPARα induces the dysregulation of a series of genes encoding mitochondrial FAO enzymes in the heart [47]. In addition, dysfunction of PGC-1α also causes abnormalities in both mitochondrial structure and function in the failing heart [48]. Consistent with these previous studies, we observed that a majority of genes associated with mitochondrial FAO, fission and fusion, and the ETC/OXPHOS are significantly decreased in *Bmal1* ^−/−^ hearts. These data support the conclusion that the reduced cardiac function observed in *H-Bmal1* ^−/−^ mice is caused by defects in the generation of mitochondrial bioenergy due to the abnormal regulation of metabolic transcription factors in the heart. In addition, we also observed that a circadian disorder imposed by the disrupted LD cycle also reduces the expression of genes involved in the ETC/OXPHOS pathway, suggesting that perturbation of overall function of the circadian clock may also induce mitochondrial defects in the heart. Of note, these changes were enhanced in PE-infused pathologic hearts compared with healthy hearts, indicating that mitochondrial function in the diseased heart is more susceptible to disorders of the circadian system.

A close association between the circadian clock and mitochondria-based cellular metabolism has been indicated in a series of studies focusing on the regulation of cellular redox. For example, the DNA binding activity of CLOCK:BMAL1 is influenced by the ratio of NAD^+^ and NADH [15] and PARP-1 (poly(ADP-ribose) polymerase 1) [49], an NAD^+^-dependent ADP-ribosyltransferase. Furthermore, CLOCK:BMAL1 is, in turn, involved in NAD^+^ synthesis by directly regulating the expression of NAMPT (nicotinamide phosphoribosyltransferase) [50], [51], the rate-limiting enzyme of the NAD^+^ salvage pathway. Consistent with these previous studies, we noted a significant decrease in NAD^+^ levels in *Bmal1* ^−/−^ hearts. The decrease in NAD^+^ levels in *Bmal1* ^−/−^ hearts may also be caused by reduced activity of mitochondrial complex I because a large portion of the oxidative conversion from NADH to NAD^+^ in the cell is undertaken by complex I. Of interest, *Bmal1* ^−/−^ hearts also showed reduced NADH levels. This observation is consistent with our gene expression analysis, which showed that a wide range of genes that are associated with TCA cycle and FAO, two major pathways that convert NAD^+^ into NADH, are downregulated in *Bmal1* ^−/−^ hearts. The reduction of both NAD^+^ and NADH levels indicates the uncoupling of the TCA cycle and ETC/OXPHOS pathway in *Bmal1* ^−/−^ hearts because the TCA cycle functions as a direct source of intramitochondrial NADH for the ETC, which, in turn, supplies NAD^+^ to the TCA cycle. Although the precise mechanisms that cause the decrease in expression of genes involved in FAO, TCA cycle, and ETC/OXPHOS in *Bmal1* ^−/−^ hearts remains to be elucidated, our data suggest that clock machinery is required to maintain cellular redox and mitochondrial function in the heart.

In conclusion, our findings that *H-Bmal1* ^−/−^ mice show mitochondrial defects in the heart, together with pathologic cardiac remodeling, indicate that the molecular clock machinery plays an important role in maintaining cardiac function by regulating mitochondrial dynamics and bioenergetics. In addition to the genetic model, we found that a circadian disorder imposed by disrupting the LD cycle also induced a striking effect on the expression levels of genes regulating mitochondrial energy metabolism, suggesting that the overall function of the internal clock, which is coupled to the external LD cycle, is essential for the maintenance of mitochondrial energy metabolism in the heart. Gaining further insight on how the molecular clock balances the capacity of mitochondrial metabolism in the heart based on the external cycle may pave new avenues to understand the pathogenesis of heart failure.

## Materials and Methods

### Mice

C57BL/6J mice bearing the modified *Bmal1* gene containing *loxP* sites [B6.129S4(Cg)-*Arntl^tm1Weit^*/J; stock number 007668] and transgenic mice expressing Cre recombinase driven by the αMHC promoter [B6.FVB-Tg(Myh6-cre)2182Mds/J; stock number 011038] were purchased from Jackson Laboratory and crossed to generate *H-Bmal1* ^−/−^ mice. We used littermate animals that harbored the floxed *Bmal1* gene but not the *Cre* transgene as controls. Both the strategy used and confirmation of the heart-specific deletion of the *Bmal1* gene are delineated in Figure S6. During the experiments, these mice were maintained on a 12∶12 LD cycle unless otherwise noted.

In all experiments in which disrupted LD cycles were applied, we used C57BL/6J mice obtained from Charles River Laboratories Japan, Inc. (Yokohama, Japan). All mice in the disrupted LD cycle group were subjected to reversals of the LD cycle every 3 days for a total 18 days. The LD reversal regimen began when the animals were 9 weeks of age. The animals in the fixed LD cycle group were exposed to a constant 12∶12 LD cycle until the end of the experiment. Osmotic pumps (ALZET model 1004, DURECT, Cupertino, CA) were used for long-term treatment with PE (30 mg/kg/day) or NS (control). Pumps were implanted subcutaneously under light anesthesia on the day when the disrupted (or fixed) LD regimen began.

In all experiments, male animals that had ad libitum access to food and water were used.

### Behavioral analysis

The locomotor activity of the animals was monitored using a Supermex system (Muromachi Kikai, Tokyo, Japan). In this system, a sensor counts the movements of the mouse, which is individually housed in a home cage, by detecting the radiated body heat. Data were recorded continuously in 1-min bins using a data collection program (CompACT AMS, Muromachi Kikai).

### Echocardiographic analysis

Transthoracic echocardiography was performed using a 15-MHz linear-array probe. The images were obtained in M-mode (left parasternal short-axis). All echocardiograms were performed blinded to the mouse genotype or condition used. LV fractional shortening was calculated using the formula (LVIDd-LVIDs)/LVIDd x 100.

### Life-span analysis

Animals for the longevity study were not used for any other physiological, biochemical, or molecular experiments. All mice for the longevity study were carefully inspected every day. The endpoint of life was when the animal was found dead during daily inspections. Moribund animals were humanely euthanized under isoflurane anesthesia by cervical dislocation upon presentation of defined criteria (diminished response to stimuli, lethargy, and failure to thrive), and the time of euthanasia was used as the endpoint. Any animals which did not meet these criteria were allowed to proceed to a natural death; however, alternative endpoints were considered when any of the following symptoms was found: 1) inability to reach food and water, 2) inability to remain upright, 3) weight loss (more than 15%). All efforts (ex. having food and water easily accessible) were made to minimize suffering of the animals. The survival data for each genotype were analyzed by plotting the Kaplan-Meier curves and performing log-rank tests.

### Tissue collection and RNA extraction

The left ventricles of hearts were used to extract total RNA. Tissues from *H-Bmal1* ^−/−^ (n = 6) and their controls (n = 6) were collected at ZT2 of the LD cycle. In the experiments employing the LD reversal regimen, heart tissues from C57BL/6J mice were obtained every 4 hours beginning at ZT2 on day 19 of the LD reversal regimen (n = 4 per group per time point). Total RNA was extracted from frozen tissue with TRIzol reagent (Invitrogen, Carlsbad, CA). The specific procedures for microarray analysis and quantitative PCR are described below.

### Microarray analysis

Heart total RNA extracted from six animals per genotype (control and *H-Bmal1* ^−/−^) was pooled and then used for a microarray analysis. We used a commercially available DNA microarray service (Takara Bio, Otsu, Japan). For this service, cyanine-3 (Cy3)-labeled cRNA was prepared from 0.5 µg total RNA. Cy3-labeled cRNA (1.65 µg) was fragmented and hybridized to the SurePrint G3 Mouse (8x60K) Microarray (Agilent Technologies, Santa Clara, CA) using standard procedures. After hybridization, the microarrays were washed with GE Wash Buffer 1 (Agilent) for 1 min at room temperature and for another 1 min with GE Wash Buffer 2 (Agilent). The microarrays were then dried by centrifugation and scanned by an Agilent DNA Microarray Scanner (G2565CA). For data processing, the scanned images were analyzed using Feature Extraction Software 10.5.1.1 (Agilent) to obtain (background-corrected and normalized) signal intensities. Gene expression levels were graphically represented in heat maps, which were created by the freely available software MultiExperiment Viewer (MeV; http://www.tm4.org/mev.html) [52].

### Quantitative RT-PCR

First-strand cDNA was synthesized using 0.25 µg of total RNA and the High Capacity cDNA Reverse Transcription Kit (Applied Biosystems, Foster City, CA). Quantitative PCR was performed and analyzed using a TP850 Thermal Cycler Dice Real-time System (Takara Bio). Samples contained 1 X SYBR Premix Ex Taq II (Takara Bio), 1000 nM of each primer, and cDNA in a 10 µl volume. The PCR conditions were as follows: 30 sec at 95°C, then 35 cycles of 5 sec at 95°C and 30 sec at 60°C. Expression levels relative to *Gapdh* were calculated using the comparative C_T_ method. The sequences of primers used for the quantitative RT-PCR are shown in Table S1.

### Histology and electron microscopy

Tissues were fixed in 4% paraformaldehyde, processed and embedded in paraffin prior to sectioning (4 microns), and stained with H&E for overall morphology and with Masson's trichrome to detect fibrosis. Heart tissues for transmission electron microscopy were cut into 1-mm pieces that were fixed immediately after collection in 2.5% glutaraldehyde in 0.1 M phosphate buffer (pH 7.4), and stored at 4°C. Post-fixation was performed in 2% OsO4 (4°C). Subsequently, samples were dehydrated and embedded in epon. Ultrathin sections were examined using a H-7100 electron microscope (Hitachi High-Technologies, Tokyo, Japan). The number of cardiac mitochondria was determined from electron microscope images. For each mouse genotype (i.e., control vs. *H-Bmal1* ^−/−^) and condition used (i.e., fixed vs. disrupted LD cycle and NS vs. PE infusion), at least 5 different images were examined in a blinded fashion.

### mtDNA quantification

To extract DNA, a small piece of heart tissue was incubated in 50 mM NaOH at 95°C and then neutralized with Tris buffer (pH 5.5). Quantitative PCR was performed using 200-fold diluted DNA, 1 X SYBR Premix Ex Taq II (Takara Bio), and 1000 nM of each primer [mtDNA-specific primers (16S rRNA): forward 5′-CCGCAAGGGAAAGATGAAAGAC-3′, reverse 5′-TCGTTTGGTTTCGGGGTTTC-3′; nDNA specific primers (hexokinase 2): forward 5′-GCCAGCCTCTCCTGATTTTAGTGT-3′, reverse 5′-GGGAACACAAAAGACCTCTTCTGG-3′] in a 10 µl volume. The PCR conditions were as follows: 30 sec at 95°C and then 40 cycles of 5 sec at 95°C and 30 sec at 56°C. Results were calculated based on differences in threshold cycle values for mtDNA and nDNA. The data are expressed as the ratio of mtDNA to nDNA copy number.

### Mitochondrial isolation and protein assay

Mitochondria were isolated from heart tissues using a differential centrifugation method following the manufacturer's protocol (BioChain Institute, Newark, CA). Briefly, 50-mg heart tissue samples were homogenized in isolation buffer. The homogenates were transferred to tubes and centrifuged at 600 *g* for 10 min at 4°C. The supernatants were again centrifuged at 12 000 *g* for 15 min at 4°C to isolate the mitochondrial pellet. The pellet was resuspended in mitochondrial isolation buffer. These centrifugation processes were then repeated once more to obtain the pure mitochondrial sample. The protein concentrations of the mitochondrial samples were determined using a Bradford protein assay kit (Nacalai Tesque, Kyoto, Japan).

### Biochemical assays

The enzymatic activities of mitochondrial complex I and IV were determined using the pure mitochondria isolated from the heart tissue. Complex I activity was analyzed using a microplate assay kit (EMD Millipore, Darmstadt, Germany). For this assay, complex I activity is measured based on the oxidation of NADH to NAD^+^, which leads to an increase in absorbance at 450 nm. The activity of complex IV was measured using the Cytochrome C Oxidase Activity Assay Kit (BioChain Institute), for which the oxidation of reduced cytochrome c at 550 nm was monitored.

### NAD^+^ and NADH measurements

The NAD^+^ and NADH levels in heart tissue were determined using the EnzyChrom NAD^+^/NADH Assay Kit (BioAssay Systems, Hayward, CA) according to the manufacturer's instructions.

### Statistical analyses

All results are presented as the means ± SEM. Survival analysis was performed using the Kaplan-Meier method, and significance was calculated based on log-rank tests. Two-way ANOVAs followed by Scheffe's post-hoc tests were used when data contained two variable factors (i.e., fixed vs. disrupted LD cycle and NS vs. PE infusion). All other comparisons were performed using unpaired two-tailed Student's *t*-tests to determine significance. In all cases, a *P* value of less than 0.05 was considered significant.

### Study approval

All animal care and use procedures were approved by the Wakayama Medical University Institutional Animal Care and Use Committee (Wakayama Medical University Permit Number: 497).

## Supporting Information

Figure S1 **Behavioral and cardiovascular rhythms are unaltered in** ***H-Bmal1*** **^−/−^ mice.** (A) Two representative actograms from control (left column) and *H-Bmal1* ^−/−^ (right column) animals. Activity counts are indicated by the vertical black marks. The records are double plotted such that each day's record is presented both to the right of and beneath that of the previous day. For the first 10 days, animals were maintained on a 12∶12 h light-dark (LD) cycle, denoted by the bar above the record. The animals were then transferred to constant darkness (DD) on the day indicated by the horizontal line at the right margin. The free-running period and the amplitude of the circadian rhythm in control and *H-Bmal1* ^−/−^ mice are shown in bar graphs (n = 8 per genotype). The free-running period was calculated as the duration of time between the major activity periods on consecutive days. The amplitude of the locomotor activity rhythm was determined using fast Fourier transformation (FFT), which estimates the relative power of the approximately 24-h periodic rhythm compared with all other periodicities. (B) Profiles of 24-h systolic and diastolic blood pressures (BPs) and heart rates (HRs) in control (black lines, n = 4) and *H-Bmal1* ^−/−^ (gray lines, n = 4) mice. Animals were maintained on a 12∶12-h LD cycle (indicated by the bar at the bottom). Each cardiovascular parameter was averaged over the 12-h light and 12-h dark periods and is expressed using a bar graph. Data are the mean ± SEM. Data were compared using unpaired two-tailed Student's *t*-test.
(EPS)

Figure S2 **Severely impaired cardiac function in 24-week-old** ***H-Bmal1*** **^−/−^ mice.** (A) Ratios of heart weight to body weight (HW/BW) at 24 weeks of age (n = 6 per group). (B) Echocardiographic analysis in 24-week-old control and *H-Bmal1* ^−/−^ animals (n = 9–12 per group). LV internal diameter at diastole (LVIDd) and at systole (LVIDs) and fractional shortening (FS) are shown in bar graph format. Data are the mean ± SEM. *P<0.05, **P<0.01, unpaired two-tailed Student's *t*-test.
(EPS)

Figure S3 **Mitochondrial abnormalities in the hearts of 24-week-old** ***H-Bmal1*** **^−/−^ mice.** (A) Representative electron micrographs of sections taken from the left ventricular muscle from control and *H-Bmal1* ^−/−^ mice at two different magnifications. (B) The mitochondrial protein concentration of control and *Bmal1* ^−/−^ heart preparations (n = 8 per group). (C) The mitochondrial DNA to nuclear DNA ratio in control and *Bmal1* ^−/−^ hearts (n = 6 per group). (D-E) Enzymatic activities of (D) complex I and (E) complex IV in control and *Bmal1* ^−/−^ hearts (n = 8 per group). The activities of these mitochondrial respiratory enzymes are expressed either per milligram of tissue used for mitochondrial isolation (top panels) or per microgram of mitochondrial protein (bottom panels). (F) NAD^+^ and NADH concentrations in control and *Bmal1* ^−/−^ hearts (n = 6 per group). Data are the mean ± SEM. *P<0.05, **P<0.01, unpaired 2-tailed Student's *t*-test.
(EPS)

Figure S4 **Circadian desynchronization not only disrupts rhythms but also reduces the expression levels of clock and metabolic genes in the hearts of NS-infused C57BL/6J mice.** (A-C) Relative expression levels of genes regulating (A) clock machinery as well as (B) glucose and (C) lipid metabolism in the heart. All heart tissues used were from the NS-infused animal group subjected to either a fixed or a disrupted LD cycle as described in Figure 5A (n = 4 per group per time point). To provide a 24-h overall mean expression level, the 24-h data were also averaged and are expressed using a bar graph format. Data are the mean ± SEM. **P<0.01, unpaired 2-tailed Student's *t*-test.
(EPS)

Figure S5 **Circadian desynchronization impairs mitochondrial function in the hearts of NS-infused C57BL/6J mice.** (A-B) Relative expression levels of genes regulating (A) mitochondrial structure and (B) mitochondrial oxidative metabolism in heart. All heart tissues used were from the NS-infused animal group subjected to either a fixed or a disrupted LD cycle as described in Figure 5A (n = 4 per group per time point). To provide overall 24-h mean expression levels, data from over a 24-h time period in each group were also averaged and are expressed using a bar graph format. (C) Enzymatic activity of complex I in NS-infused animals exposed to a fixed or disrupted LD cycle (n = 8 per group). The complex I activity is expressed per milligram of tissue used for mitochondrial isolation. (D) The relative number of mitochondria in the left ventricular muscle was counted using electron microscope images. Representative images are shown. Data are the mean ± SEM. *P<0.05, unpaired two-tailed Student's *t*-test.
(EPS)

Figure S6 **Heart-specific disruption of the** ***Bmal1*** **conditional allele.** (A) Scheme showing the conditional knockout of exon 8, which encodes the basic helix-loop-helix domain of the BMAL1 protein. Triangles, *loxP* sites; bars with base pair (bp) markers, sites and sizes of PCR products diagnostic of the heart-specific disruption of the *Bmal1* gene. (B) Confirmation of the heart-specific deletion of exon 8 in the *Bmal1* gene of *H-Bmal1* ^−/−^ mice. PCR products amplified from genomic DNA, which were extracted from the heart tissue of a control mouse (homozygous *Bmal1* conditional, no *Cre*) and from the heart, lung, and kidney tissues of an *H-Bmal1* ^−/−^ mouse (homozygous *Bmal1* conditional, *Myh6Cre*).
(EPS)

Table S1 Primer sequences used for quantitative RT-PCR.
(DOCX)

Materials S1 **Materials and methods for analysis of cardiovascular parameters.**
(DOCX)
