# Supplementary material for: The Circadian Clock Maintains Cardiac Function by Regulating Mitochondrial Metabolism in Mice
Source: PLoS One. 2014 Nov 12;9(11):e112811. doi: 10.1371/journal.pone.0112811 (PMC4229239; doi:10.1371/journal.pone.0112811)
Supplement: Table S1 — Primer sequences used for quantitative RT-PCR. (DOCX) [file pone.0112811.s007.docx]

**Table S1. Primer sequences used for quantitative RT-PCR**

| Gene name | Sequences |
| --- | --- |
| *Gapdh* | 5'-CAAGGAGTAAGAAACCCTGGACC-3' |
|  | 5'-CGAGTTGGGATAGGGCCTCT-3' |
| *ANP* | 5'-AGAGACGGCAGTGCTCTAGG-3' |
|  | 5'-AGCCCTCAGTTTGCTTTTCA-3' |
| *BNP* | 5'-CACCCAAAAAGAGTCCTTCG-3' |
|  | 5'-GCCCAAAGCAGCTTGAGATA-3' |
| *Cpt2* | 5'-CGGCCCTTAAGTGCTGTCT-3' |
|  | 5'-AGGCTGTCCTGGTAGTGCAT-3' |
| *Acsl1* | 5'-TGATCCAGAAGGGGTTCAAG-3' |
|  | 5'-GAGAAGAGGCCGATGAACTG-3' |
| *Fabp3* | 5'-AGGTGGCTAGCATGACCAAG-3' |
|  | 5'-TATCCCCGTTCTTCTCGATG-3' |
| *Ehhadh* | 5'-TAAACAAGCCAGTCCCAAGC-3' |
|  | 5'-TGGCTTCTGCAAAAACACTG-3' |
| *Hadha* | 5'-CCCAAGAAGCAACACGAATA-3' |
|  | 5'-CTCAAGTCCTCCTCCCAAGC-3' |
| *Pgam1* | 5'-CATTGACCAGATGTGGTTGC-3' |
|  | 5'-TGTCAGACCGCCATAGTGTC-3' |
| *Pdk4* | 5'-GACCCCGTTACCAATCAAAA-3' |
|  | 5'-GTGCTGATTCATGAGCATCC-3' |
| *Ogdh* | 5'-GCACAACCTAACGTCGACAA-3' |
|  | 5'-ATGCCCTGATGAGAGACTGC-3' |
| *Idh3b* | 5'-CGTGATGCCCAATCTCTATG-3' |
|  | 5'-CACTGTAGCTCTCCCCAGGA-3' |
| *Mdh2* | 5'-GAAGAACAGCCCCCTAGTGA-3' |
|  | 5'-GACTCAGATCTGCTGCCACA-3' |
| *Ndufs7* | 5'-CCCAAGCTCTCTCATCTTCC-3' |
|  | 5'-TCCAGCTTGGTCACCACATA-3' |
| *Sdhc* | 5'-TGTGAAGTCCCTGTGTTTGG-3' |
|  | 5'-AAGTGTCGGATCCCATTCAG-3' |
| *Uqcrc1* | 5'-TGCTGAGGTTACCTGCCTTG-3' |
|  | 5'-TTGTCCAAGATGCTGACCTG-3' |
| *Cox7b* | 5'-AGGGTAGCTGGGGTGAATTT-3' |
|  | 5'-TTTTGGCTAAGGGCAACATC-3' |
| *Apt5g2* | 5'-TTTCAAGGGACATCGACACA-3' |
|  | 5'-TCCCAAAAACAGTCCCAATC-3' |
| *Ppargc1a* | 5'-TTGCTAGCGGTTCTCACAGA-3' |
|  | 5'-AAATGAGGGCAATCCGTCTT-3' |
| *Ppara* | 5'-TGCAAACTTGGACTTGAACG-3' |
|  | 5'-AGGAGGACAGCATCGTGAAG-3' |
| *Mfn1* | 5'-TGCAATGCTGTGGGATAAAG-3' |
|  | 5'-TCAGGAAGCAGTTGGTTGTG-3' |
| *Mfn2* | 5'-GGAAGAGCACCGTGATCAAT-3' |
|  | 5'-CAATCCCAGATGGCAGAACT-3' |
| *Opa1* | 5'-CTTTTGGCCAGCAAGGTTAG-3' |
|  | 5'-GGCTGTATAGCCACCTCCAA-3' |
| *Ucp2* | 5'-GCGTTCTGGGTACCATCCTA-3' |
|  | 5'-ATTGTAGAGGCTGCGTGGAC-3' |
| *Bcl2* | 5'-AAGCTGTCACAGAGGGGCTA-3' |
|  | 5'-CAGGCTGGAAGGAGAAGATG-3' |
| *Per2* | 5'-TGTGCGATGATGATTCGTGA-3' |
|  | 5'-GGTGAAGGTACGTTTGGTTTGC-3' |
| *Bmal1* | 5'-CCACCTCAGAGCCATTGATACA-3' |
|  | 5'-GAGCAGGTTTAGTTCCACTTTGTCT-3' |
| *Rev-erbα* | 5'-ATGCCCATGACAAGTTAGGC-3' |
|  | 5'-GGGCTACCTGATGCATGATT-3' |
| *Glut1* | 5'-ACTCACCACGCTTTGGTCTC-3' |
|  | 5'-ACAAAGAGGCCGACAGAGAA-3' |
| *Glut4* | 5'-GACGCACTAGCTGAGCTGAA-3' |
|  | 5'-ATTGGACGCTCTCTCTCCAA-3' |
| *Drp1* | 5'-AGAGCTCAGTGCTGGAAAGC-3' |
|  | 5'-CACCAGTTCCTCTGGGAAGA-3' |
| *Ndufa1* | 5'-CGGAGATGTGGTTCGAGATT-3' |
|  | 5'-CGTTGGTGAATTTGTGGATG-3' |
| *Ndufv1* | 5'-TGACTGGATCTTGGGTGAGA-3' |
|  | 5'-TCATGAAGCTCCACTTGAGG-3' |
| *Sdha* | 5'-TTGCGGGAAGGCAGACAT-3' |
|  | 5'-CGAAGAAGCCTCGAAACTG-3' |
| *Uqcrq* | 5'-GATCTACACATGGGGCAACC-3' |
|  | 5'-GCAGGCCGTCTACTTGTCAT-3' |
| *Cyc1* | 5'-TAGCTGTGGCCCTTCATTCT-3' |
|  | 5'-ACGATGAGACCATGGGTAGC-3' |
