# Supplementary material for: The Circadian Clock Maintains Cardiac Function by Regulating Mitochondrial Metabolism in Mice
Source: PLoS One. 2014 Nov 12;9(11):e112811. doi: 10.1371/journal.pone.0112811 (PMC4229239; doi:10.1371/journal.pone.0112811)
Supplement: Materials S1 — Materials and methods for analysis of cardiovascular parameters. (DOCX) [file pone.0112811.s008.docx]

**Materials S1**

***Analysis of cardiovascular parameters.***

Arterial blood pressure and heart rate were telemetrically monitored in conscious, freely moving 12-week-old mice using a pressure-sensing catheter connected to a battery-operated transmitter (PA-C10, Data Science International, St Paul, MN). Under anesthesia [a mixture of ketamine (100 mg/kg, i.p.) and xylazine (10 mg/kg, i.p.)], the catheter was inserted into the aortic arch via the common carotid artery, and the transmitter was then placed in a subcutaneous pouch along the animal's flank. After 1 week of recovery period, data were collected via a receiver (RPC-1; Data Science International). The data collected were stored and analyzed using a computerized system (PowerLab/8s; ADInstruments Japan, Inc., Nagoya, Japan) to determine 24-h diurnal variations in systolic and diastolic blood pressures and heart rate. These cardiovascular parameters were monitored for the first 30 sec of every 5 min for at least 2 days. Results at each time point represent an average over 2 consecutive days.
